# Sensor Classification Using Convolutional Neural Network by Encoding Multivariate Time Series as Two-Dimensional Colored Images

**DOI:** 10.3390/s20010168

**Published:** 2019-12-27

**Authors:** Chao-Lung Yang, Zhi-Xuan Chen, Chen-Yi Yang

**Affiliations:** Department of Industrial Management, National Taiwan University of Science and Technology, Taipei City 10607, Taiwan; a2265848@gmail.com (Z.-X.C.); cathy19931008@gmail.com (C.-Y.Y.)

**Keywords:** time series classification, multivariate time series, image concatenation, convolutional neural network

## Abstract

This paper proposes a framework to perform the sensor classification by using multivariate time series sensors data as inputs. The framework encodes multivariate time series data into two-dimensional colored images, and concatenate the images into one bigger image for classification through a Convolutional Neural Network (ConvNet). This study applied three transformation methods to encode time series into images: Gramian Angular Summation Field (GASF), Gramian Angular Difference Field (GADF), and Markov Transition Field (MTF). Two open multivariate datasets were used to evaluate the impact of using different transformation methods, the sequences of concatenating images, and the complexity of ConvNet architectures on classification accuracy. The results show that the selection of transformation methods and the sequence of concatenation do not affect the prediction outcome significantly. Surprisingly, the simple structure of ConvNet is sufficient enough for classification as it performed equally well with the complex structure of VGGNet. The results were also compared with other classification methods and found that the proposed framework outperformed other methods in terms of classification accuracy.

## 1. Introduction

In the era of data explosion, time series data, which is a series of data points indexed in time order, is one of the most common data collected. A variety of time series data can be collected from the internet, machines, devices, and sensors for all kinds of applications such as monitoring, tracking, and pattern classification. Multivariate time series (MTS) data from multiple resources can be used to present the operating statuses of the machines, or human health condition such as electrocardiography. In smart manufacturing, building a binary classification model by machine learning algorithm to identify defects or tool wearing (normal or abnormal) from the collected time series data is also a popular approach to improve production quality [1].

Assume a time series x is a set of data points indexed in time order, x={x(t)∈R:t=1,2,…,T}, where T represents the length of the time series data [2]. Górecki and Łuczak defined an MTS X as multiple univariate time series, such that $X=\left(x_{1},x_{2},\ldots ,x_{m}\right)$, $x_{i}=\left\{x_{p}\left(t\right)\in R:t=1,2,\ldots ,T\right\}\;\left(p=1,2,\ldots ,m\right)$, where m is the number of univariate time series in X that also represents the dimension. 

An MTS can be considered as a m×n matrix. Generally, MTS data mining research can be categorized into: (1) representation and indexing, (2) similarity measure, (3) segmentation, (4) visualization, and (5) mining [3]. Essentially, MTS classification belongs to a “mining” area that tries to categorize multiple time series as class labels [4]. There have been several challenges in dealing with high-dimensional data for MTS classification. For instance, a univariate time series usually includes a lot of noise in the process of collecting data. The noise issue is compounded in MTS, which is composed of multiple univariate time series. Another challenge is MTS classification tools have to not only recognize the data features but also consider the correlations among the variables. 

In the early years, traditional time series analysis techniques such as: Simple Exponential Smoothing (SES) [5], Autoregressive Integrated Moving Average (ARIMA) [2], and dynamic time warping (DTW) method [6], which were used for analyzing MTS data for measurement or estimation. Combining with different measurement techniques, multiple machine learning methods such as decision tree [7], Support Vector Machine (SVM) [8], neural network [9], and so on have been proposed to solve the MTS classification problem. 

Lately, with the maturity of deep learning technology and advances in Graphics Processing Unit devices, many studies used deep learning frameworks to address MTS classification problems. Fawaz et al. reviewed multiple deep learning methods and specified an overview of the different deep learning approaches for time series classification [10]. In their review, deep learning approaches for MTS classification can be categorized as two main models: the generative and the discriminative models. By exhibiting an unsupervised training step to find a good representation of time series that precedes the learning phase of the classifier, generative models were considered as model-based classifiers [11]. On the other hand, a discriminative deep learning model directly learns the mapping between the inputs of MTS and class outputs with the feature engineering and model tuning. To avoid the ambiguity, in this research, we focus on developing a discriminative deep learning model based on image-based time series data representation, considered as an innovative preprocessing of feature engineering.

Among versatile deep learning models, Convolutional Neural Network (ConvNet) has gained rapid adoption. This method can extract more features and details from the input image, classify and predict classes more precisely than previous machine learning algorithms. Researchers had applied it to solve problems in different fields, of course that includes MTS classification (TSC) problems. By directly using time series data as inputs, Zheng et al. proposed supervised feature learning with ConvNet to classify time series data [12]. Gamboa also adopted ConvNet for time series analysis and obtained promising results [13]. Yazdanbakhsh and Dick used same size segments in time series with sliding window to transform an image and applied Dilated Convolutional Neural Network for classification [14]. 

Instead of using raw time series data as input, Wang and Oates first encoded univariate time series data into different types of images, namely, Gramian Angular Fields (GAF) and Markov Transition Fields (MTF), as inputs of ConvNet [15,16]. In fact, this image-based framework initiated a new branch of deep learning approaches which consider image transformation as one of feature engineering technique. The transformation of time series into images was inspired from the computer vision feature extraction concept. By learning spatially invariant filters (or features) from raw input time series, ConvNet method diminished the problems of: (1) temporal information is lost and (2) the features learned are no longer time-invariant which are with the traditional multi-layer-perceptron approach. In their most recent work, Chen and Shi followed the same framework adopting Relative Position Matrix with ConvNet, called RPMCNN, to perform the classification by the transform 2D images from time series data as inputs [17]. Their results all showed promising performances by converting univariate time series data to 2D images as an input of ConvNet.

Although the result of encoding time series data as image representation for ConvNet is promising in improving classification accuracy, most of the previous works only considered encoding univariate time series data as one image for a single channel of ConvNet’s input. In other words, when MTS data are collected, how to combine the information of MTS image as inputs of ConvNet is still an open domain. In addition, for an MTS classification problem, the framework of considering colors of MTS images for ConvNet is needed. Therefore, in this research, we proposed an innovative framework which transforms one batch of MTS data into multiple images and concatenating them as bigger two-dimensional images as inputs of ConvNet. The deep learning architecture of ConvNet was then applied to extract and learn features from these images for classification purpose. Three typical methods of encoding MTS data into images, the sequences of image concatenation and two kinds of ConvNet architectures were investigated. Two open multivariate datasets which are the benchmarks datasets were used to evaluate the experiment results. The results show that proposed framework can enhance the accuracy of MTS classification by using the relatively simple network. In short, we conclude this work as the followings:

- This work aims to extend 2-D image transformation method for MTS classification from the univariate time series input to MTS inputs;
- The proposed innovative image concatenation can combine MTS data as multiple color channels as inputs of ConvNet;
- The proposed framework can enhance the accuracy of MTS classification by using the relatively simple network;
- The result shows the selection of image transformation methods and the sequence of image concatenation are not significant for classification accuracy.

The rest of the paper is organized as follows: Section 2 provides a review of MTS, data encoding methods, and ConvNet; Section 3 describes the methodologies of data transformation, image aggregation, and ConvNet hyperparameter setting; Section 4 explains the experiments and results; and Section 5 presents the conclusion and suggestions for future research. 

## 2. Literature Review

### 2.1. Convolutional Neural Network (ConvNet)

In recent years, ConvNet is widely used as the deep learning algorithm for computer vision to detect meaningful features and patterns. The concept of this framework was introduced by two neurophysiologists, Hubel and Wiesel, who were inspired by the visual cortical neurons of cats and monkeys. However, the first researchers who used backpropagation applied in ConvNet were LeCun et al., who also started the new era of ConvNet [18]. Over time, many outstanding architectures were developed, such as the AlexNet [19], VGGNet [20], ResNet [21], and Inception v3 [22]. They achieved good results in the ImageNet Large Scale Visual Recognition Competition (ILSVRC) each year.

A typical ConvNet consists of a convolutional layer, an activation function, a pooling layer, a fully connected layer, and an output layer. The convolutional layer extracts meaningful features such as edges, color, and gradient orientation, from the input image by using linear function. The output matrix is the result of computing the dot product when a filter covers the input image.

Activation functions plays a non-linear role between the convolutional layer and pooling layer in a ConvNet model. The Rectified Linear Unit (ReLU) is the popular activation function in the last few years in the deep learning field [23], although this concept was proposed as early as the year 2000 [24]. The advantages of ReLU are it reduces the vanishing gradient problem and allows models to learn faster and perform better.

The main purpose of the pooling layer is to reduce the spatial dimensions of the feature map but still preserve the important information. Generally, the feature map is shrunk by a factor greater than or equals to two. The max pooling method [25], which simply calculates the maximum value of each patch in the feature map, is often used in the pooling layer. After passing through multiple convolutional and pooling layers, the output is converted into a dense vector by flattening the pooled feature map from two dimensions to one dimension. Lastly, a ConvNet uses the feedforward neural network to compute the different weights between nodes, and get the probabilities of different classes.

### 2.2. Image Based Time Series Data

Due to the rapid developing of computer vision, the idea of classifying time series data using computer vision technology was inspired. Various transformation methods were proposed to encode times series as input images of computer vision, in hope that the two-dimensional images can reveal features and patterns not found in the one-dimensional sequence of the original time series. 

Two of the popular data transformation methods are the Gramian Angular Field (GAF) and the Markov Transition Field (MTF) [15]. GAF encodes time series into image by polar coordinates based matrix and it can preserve absolute temporal correlation [26]. The original time series x is first normalized to between 0 and 1, which is defined in Equation (1).

(1) $${\tilde{x}}_{0}^{t}=\frac{x\left(t\right)-min\left(x\right)}{max\left(x\right)-min\left(x\right)}$$

Then, angular cosine and the time stamp are used to encode the rescaled data into polar coordinates. From top-left to bottom-right, the image position corresponds to the raw time series and it is symmetrical by the main diagonal. Due to this characteristic, the polar coordinates can revert back to the raw time series by its transformation principle. GAF can generate two images by different equations. The Gramian Angular Summation Field (GASF) is defined in Equations (2) and (3) and the Gramian Angular Difference Field (GADF) is defined in Equations (4) and (5). The difference is the conversion of trigonometric functions, where GASF is based on cosine functions and GADF is based on sine functions.

(2) $$\mathrm{GASF}=\left[\begin{array}{ccc} \cos\left(\emptyset_{1}+\emptyset_{1}\right) & \cdots & \cos\left(\emptyset_{1}+\emptyset_{n}\right)\\ \cos\left(\emptyset_{2}+\emptyset_{1}\right) & \cdots & \cos\left(\emptyset_{2}+\emptyset_{n}\right)\\ \vdots & \ddots & \vdots \\ \cos\left(\emptyset_{n}+\emptyset_{1}\right) & \cdots & \cos\left(\emptyset_{n}+\emptyset_{n}\right) \end{array}\right]$$

(3) $$\mathrm{GASF}={\tilde{x}}^{\prime }\cdot \tilde{x}-{\sqrt{I-{\tilde{x}}^{2}}}^{\prime }\cdot \sqrt{I-{\tilde{x}}^{2}}$$

(4) $$\mathrm{GADF}=\left[\begin{array}{ccc} \sin\left(\emptyset_{1}+\emptyset_{1}\right) & \cdots & \sin\left(\emptyset_{1}+\emptyset_{n}\right)\\ \sin\left(\emptyset_{2}+\emptyset_{1}\right) & \cdots & \sin\left(\emptyset_{2}+\emptyset_{n}\right)\\ \vdots & \ddots & \vdots \\ \sin\left(\emptyset_{n}+\emptyset_{1}\right) & \cdots & \sin\left(\emptyset_{n}+\emptyset_{n}\right) \end{array}\right]$$

(5) $$\mathrm{GADF}={\sqrt{I-{\tilde{x}}^{2}}}^{\prime }\cdot \tilde{x}-{\tilde{x}}^{\prime }\cdot \sqrt{I-{\tilde{x}}^{2}}$$

MTF uses Markov transition probabilities to maintain details in the time domain [15]. MTF is composed of Markov transition probabilities $M_{ij}$ of quantile bin $q_{i}$ moves to $q_{j}$, at time stamp i and j, respectively. Suppose a time series x=x(1),x(2),…,x(T), and quantile $Q=q_{1},q_{2},\ldots ,q_{j}$. The size of Q affects the Markov transition matrix (w) size. MTF is defined in Equation (6).

(6) $$M_{ij}=\left[\begin{array}{ccc} w_{ij}|x\left(1\right)\in q_{i},x\left(1\right)\in q_{j} & \cdots & w_{ij}|x\left(1\right)\in q_{i},x\left(n\right)\in q_{j}\\ w_{ij}|x\left(2\right)\in q_{i},x\left(1\right)\in q_{j} & \cdots & w_{ij}|x\left(2\right)\in q_{i},x\left(n\right)\in q_{j}\\ \vdots & \ddots & \vdots \\ w_{ij}|x\left(n\right)\in q_{i},x\left(1\right)\in q_{j} & \cdots & w_{ij}|x\left(n\right)\in q_{i},x\left(n\right)\in q_{j} \end{array}\right]$$

MTF can preserve details in the temporal range. However, as the transformed matrix is formed by the probabilities of element moving, the MTF method cannot revert to the raw time series data like GAF. In addition, as MTF is formed by the probabilities of element moving, it is not as symmetrical as GAF method. For both GAF and MTF, the transformed values can be represented as colors via the colormap. The colormap contains the colors of a rainbow. The redder color corresponds to a larger value and the bluer color corresponds to a smaller value. 

Both GAF and MTF were applied in many studies. For example, Mitiche, et al. utilized GAF in an Electromagnetic Interference (EMI) image study for extracting significant information [27]. In their work, the GAF method was combined with two feature reduction methods called the Local Binary Pattern and the Local Phase Quantization to remove redundancy. The Random Forest method was implemented to classify the images with promising outcomes. In addition, Sánchez and Cervera used electrocardiogram (ECG) data from the PhysioNet/CinC Challenge 2017 to detect atrial fibrillation [28]. The data was encoded into GASF and fed into a feed forward neural network and ConvNet for classification. Similarly, Nagem et al. encoded the American Geostationary Operational Environmental Satellite (GOES) data into MTF images, and applied ConvNet to predict the status of solar flares [29]. In the field of financial technology, Chen et al. proposed the mean average mapping method and the double moving average mapping method to encode the time series into two-dimensional images, and compare them with the GAF method [30]. The images of the mentioned methods were fed into ConvNet, and the results showed that the GAF outperforms the others.

**Figure 1:**
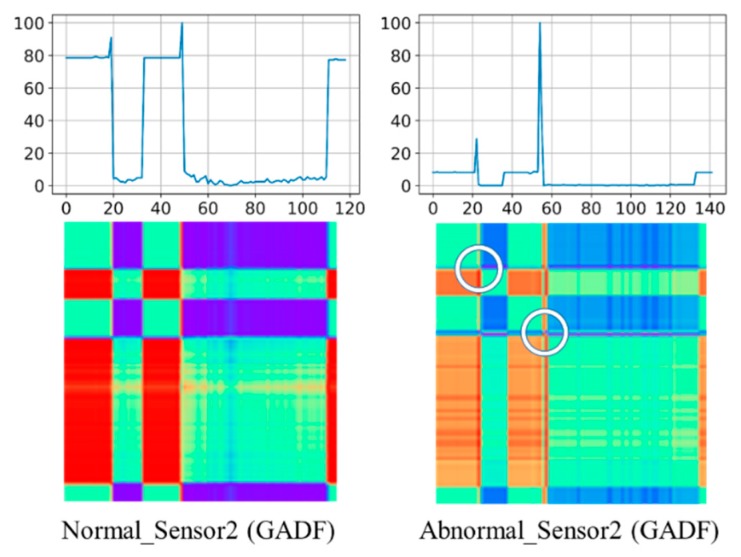
A Gramian Angular Difference Field (GADF) example of normal sensor and abnormal sensor in a Wafer dataset.

To illustrate the advantage of transforming time series data into two-dimensional images, Figure 1 shows an example of the comparison between normal and abnormal sensor data under GADF transformation from the Wafer dataset [31]. In the Wafer dataset, each time series is labelled as abnormal or normal for identifying whether the wafer process has defect. The left side of Figure 1 shows the normal time series’ sensor data and the corresponding GADF images while the right side of Figure 1 shows abnormal case. As can be seen, the abnormal time series has relatively low values and two obvious spikes comparing with the normal one. The corresponding GADF image of the abnormal case can be easily recognized that it has relatively lighter color with two distinct crossing lines (marked by the white circles) to represent the two spikes. Therefore, the characteristics of time series data can be identified in two-dimensional image from different features such as color, points, and lines at the corresponding locations in the image.

**Figure 2:**
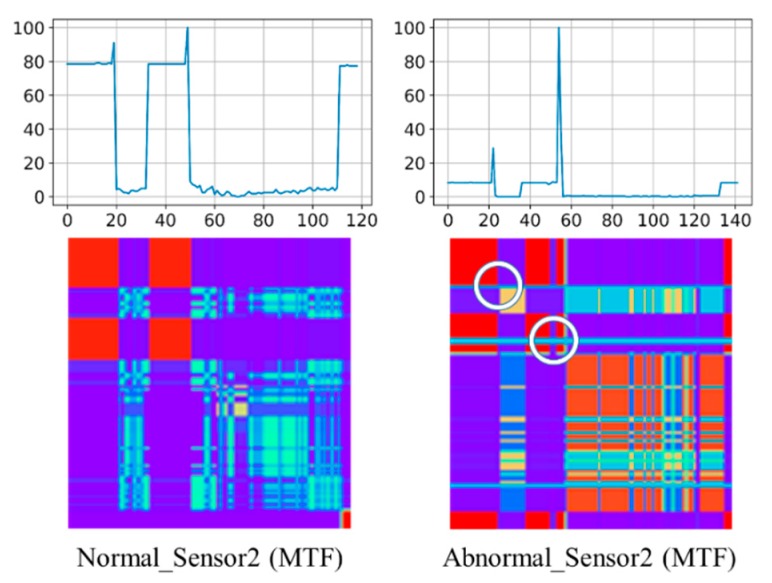
A Markov Transition Field (MTF) example of normal sensor and abnormal sensor in a Wafer dataset.

Similarly, Figure 2 shows an MTF example of the comparison between normal and abnormal sensor data (the same as time series data in Figure 1) from the Wafer dataset. As can be seen similarly, the abnormal case shown on the right hand side can be recognized with different color mapping and unique cross-lines due to the relatively high values (marked by the white circles) representing the two spikes. Although GAD and MTF shares this similarity, it is interesting to evaluate which transformation can perform better in terms of classification accuracy.

## 3. Methodology

**Figure 3:**
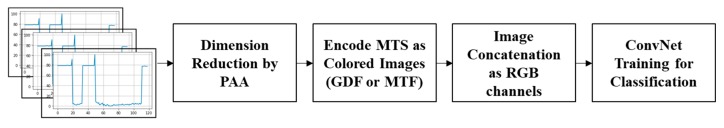
A workflow of the proposed framework for MTS Classification by Convolutional Neural Network (ConvNet).

This research is to propose a framework to classify MTS data using deep learning technology. This study first applied MTF, GASF, and GADF to transform MTS data into images. Then, the transformed images were concatenated for processing by ConvNet to identify features in the images for classification. Basically, this framework consists of four steps: (1) dimension reduction of time series, (2) image encoding, (3) image concatenation, and (4) ConvNet classification model training. Figure 3 shows the workflow of the proposed framework for MTS Classification by ConvNet. The details of this framework are introduced in the following sub-sections.

### 3.1. Dimensionality Reduction Using Piecewise Aggregate Approximation (PAA)

An image is composed of pixels, so it can be considered as a n×n matrix, where *n* defines the image size. When the length of the time series data is *n*, the image size of any kind of transformation method is n×n [26]. As each batch of time series data can vary in length, the straight transformation of the original data into images will result in different sizes of images. Therefore, to obtain images of the same size for ConvNet, in this research, Piecewise Aggregate Approximation (PAA) method is applied to perform dimension reduction of the original time series data before transforming time series data into images [32]. Please note that applying PAA is also the convention method for data preprocessing before transferring time series to images [17]. 

PAA divides original time series into *N* equal-length segments. *N* is the length of the reduced times series that should satisfy the constraint of 1 ≤ *N* ≤ *T*. Then, the mean value of each segment substitutes the original time series to reduce the dimensionality from *T* to *N*. Suppose a time series x=x(1),x(2),…,x(T) where T is the length of the original time series. *T*/*N* denotes as the length of each segment. It also means the original time series *x* will be divided by *N* segments and the reduced time series can be denoted as $\bar{x}=\left\{\bar{x\left(l\right)}\in R:l=1,2,\ldots ,N\right\}$ based on Equation (7) where *l* is the index of the reduced time series. If N=1, $\bar{x}$ is the mean of the original time series; If N=T, $\bar{x}$ is the original time series. In this research, in order to synchronize the image size, *N* is determined by the shortest length of MTS. Inevitably, the information losing on the longer timer series occurs. Although PAA will reduce the dimensionality of some time series, the result shows the classification can be improved based on concatenating multiple time series. The more detailed information can be found in Section 4.

(7) $$\bar{x\left(l\right)}=\frac{N}{T}\overset{\frac{T}{N}l}{\underset{k=\frac{T}{N}\left(l-1\right)+1}{\sum }}x_{k}$$

### 3.2. Time Series Data Encoding As Images

In this study, a 3-dimensional matrix is formed to contain the MTS. First, a time series data is encoded as a color image which has two dimensions using the GDF or MTF method. As the image can be of any color, adding one more dimension to represent the color is required. For example, the image can be represented with 3 color channels by red, green, and blue (RGB). Then, 3 elements in the first dimension exists. Please note that more colors can be used for representing more color channels. In this work, only RGB channels were to evaluate the concept of the framework. 

### 3.3. Image Concatenation

**Figure 4:**
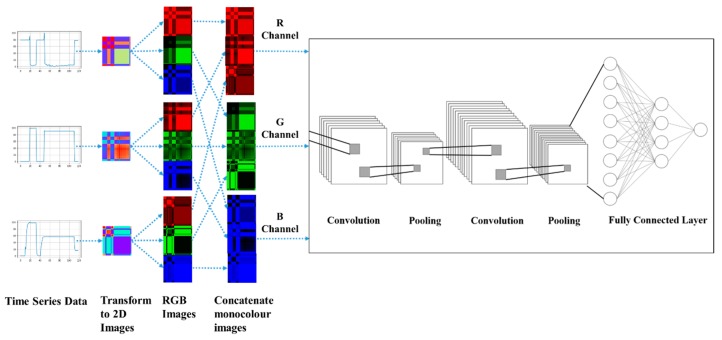
Illustration of the concept of the concatenating method with red, green, and blue (RGB) images, if three time series are considered.

MTS data transformation produces multiple images (one image for each univariate time series). These images have to be combined before feeding the ConvNet. This study adopted the concatenating method proposed by Yang et al. [33]. For RGB image aggregation, each colored image was first separated into three monochroic images: red, green, and blue (RGB) in this case. Then these monocolor images were concatenated together as a bigger image. Figure 4 illustrates the framework of concatenating RGB images. Please note that if more time series data are used as inputs for classification, more 2D images will be generated accordingly. However, only three RGB channels will be constructed in this case. Basically, this design is to maintain the same number of the input channels of the network structure which will benefit on keeping the ConvNet network structure simple. This design is particularly convenient to apply on the domains such as anomaly detection where the time series data can be processed on the edge computing from a variety of sensors, and the image files can be uploaded as inputs of ConvNet which might be in the different location such as on cloud computing environment.

There is an interesting issue regarding the “spurious edge” created by concatenating 2D images. The question is if the “spurious edge” influences the classification? In order to study this issue, an experiment was designed to evaluate the sequence of concatenating 2D images. The concatenated images with different sequence of the 2D images (the different patterns of “spurious edges”) are compared with their classification performance. The experimental result shows the patterns of “spurious edges” will not significantly influence the classification result. The details of this experimental results can be found in Section 4.

### 3.4. The Architecture of a ConvNet

In this study, for each time series data, the size of 2D transformed image is fixed at 128×128 pixels. Due to the nature of the proposed concatenation method, if *m* time series exists, the size of the input image for the ConvNet is fixed at (128×m)×128 for each monochrome channel. For RGB images, three channels will be allocated. 

In order to assess whether the complexity of ConvNet architecture affects the classification accuracy, in this research, two kinds of ConvNet, noted as the simple ConvNet and VGG16, are studied. VGG16 proposed by Simonyan and Zisserman is the model won the ImageNet Large Scale Visual Recognition Competition (ILSVRC) in 2014 [20]. 

For the simple ConvNet, we adopted the very popular model devised by Palm [34]. Two convolutional layers with a kernel size of 5×5, two max pooling layers with a 2×2 pixel window and stride of 2, and one fully-connected layer are suggested. After max pooling, the height and width of the input image becomes half. The learning rate was set to 0.0023 and the rectification non-linearity was applied to all hidden layers as the activation function based on the setting suggested in [19]. To prevent the overfitting problem, the early stopping method was implemented according to the suggestion in [35]. This method can also reduce memory and decrease computation time.

Because VGGNet uses more layers and smaller size of convolutional filters to construct the deeper depth of network structure, in this work, we consider VGGNet as a larger network for learning which is expected to classify images more accurately. This research adopted the typical VGG16, which has 13 convolutional layers with a kernel size of 3×3, 5 max pooling layers with a 2×2 pixel window and 3 fully-connected layers. The learning rate was set to 0.00023 based on [20]. Most of the learnable parameters are used in the first fully-connected layers. The number of learnable parameters in VGG16 is 201,330,688, which is 800 times larger than the simple ConvNet (251,542). Obviously, VGG16 can be expected to spend more execution time and memory than typical ConvNet.

## 4. Experiments and Results

In this work, three series of experiments were conducted to evaluate the impact of: (1) the image transformation methods, (2) the sequences of concatenating images, and (3) the structure complexity of the network. As mentioned earlier, the first experiment was to evaluate the significance of utilizing image transformation methods: GASF, GADF, and MTF methods as inputs of ConvNet. The second experiment aimed to study the impact of “spurious edges” which are generated by concatenating images. The different sequences of concatenating 2D images were evaluated to check if the classification performance was affected by the sequence, or “spurious edges” of concatenated images. The performances of different random sequences are compared with each other. The third experiment focused on evaluating if the more complicated network structure is able to further improve the classification accuracy.

The MTS data were transformed by three methods (GASF, GADF, and MTF) using the pyts package [36]. All experiments were carried out in Python 3.6 coding environment. The deep learning frameworks were built in PyTorch 1.1. The tests were conducted on a computer with Intel^®^ Core I7-8700K CPU 3.7 GHz, 64GB RAM, GeForce GTX Titan Xp video card, and Windows 10.

### 4.1. Introduction of Data Set

In this study, two popular MTS datasets, benchmark datasets for binary classification of MTS data, were used to evaluate the performance of the proposed framework. The Wafer dataset was collected from six vacuum chamber sensors that monitored the manufacture of semiconductor microelectronics. The ECG dataset in which exactly one heart beat exists per series was collected from two electrodes that recorded heartbeats as normal or abnormal. Both of the datasets were provided by Olszewski [31] and the classes of both datasets are binary (normal or abnormal). The details of these two datasets are described in Table 1.

The data length can be different in each batch, but within the same batch, the data length is the same for all sensor variables. As the range of values collected by multiple sensors is different, the data were normalized to between 0 and 1. Then the data were smoothed using the PAA mentioned in Section 3 before transformation into images.

### 4.2. Performance Evaluation

Five-fold cross validation was applied to avoid overfitting problem. It also means for each fold, 80% of the data was used for training the simple ConvNet and VGG16 while the remaining 20% was used to test the deep learning tools. The accuracy rate and the error rate are the common measures to evaluate the performance of a classification tool. Equation (8) shows the formula to calculate the error rate. When the predicted class is the same as the actual class, the value of correct is 1, or 0 otherwise. N is the total number of testing data in each dataset.

(8) $$\mathrm{Error}\;\mathrm{rate}=1-\frac{\sum_{i=1}^{n}correct_{i}}{N}\times 100\%$$

### 4.3. Experimental Results

In this research, three experiments were conducted. Each experiment used the five-fold cross validation and ran for 20 times to obtain the mean value of error rate. The first experiment investigated the impact of image transformation method GASF, GADF, and MTF under the proposed RGB image concatenation using the simple ConvNet. The second experiment evaluated the impact of the sequence of concatenating images. The third experiment explored whether the more complex architecture of the ConvNet can produce better classification results.

#### 4.3.1. Experiment #1: Comparison of Image Transformation Method

**Figure 5:**
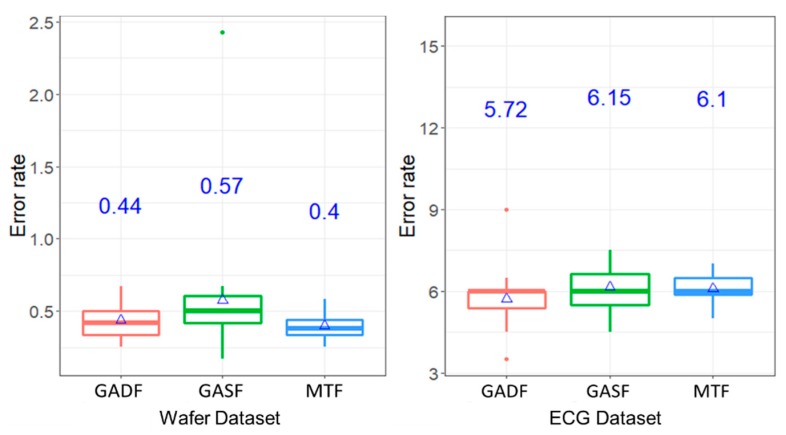
The boxplot of error rates of classification by using RGB images as inputs by GADF, Gramian Angular Summation Field (GASF), and MTF transformation methods (Wafer and electrocardiogram (ECG) database).

Figure 5 shows the boxplot of the average error rates by classifying classes of Wafer dataset under RGB images inputs of ConvNet. As mentioned earlier, three image encoding methods: GADF, GASF, and MTF were used. As can been seen, the mean error rates, indicated in the blue ink on the center of the plot, are between 0.4% and 0.57% for Wafer dataset. Similarly, the average error rates by the case of ECG dataset are between 5.72% and 6.15%. 

Further statistical analysis, through the Dunn tests, was conducted to determine whether different image transformation methods affect the error rates. Based on the results presented in Table 2, the error rates are not significantly different among pairwise comparison of the three methods in the ECG dataset under 95% confidence interval. Although the mean error rates of GASF and MTF, which are the largest and lowest in the Wafer dataset, respectively, are significantly different, the pairwise comparisons between GASF and GADF, and between GADF and MTF are not significant. In short, the selection of the image transformation seems not to affect the classification result in terms of error rates. 

#### 4.3.2. Experiment #2: Comparison of Different Sequences of Concatenating Images

In this experiment, only Wafer dataset was used because ECG has only two time series which cannot represent the complication of different image concatenation. In the Wafer dataset, each batch contains data collected from six sensors. Hence, the transformed images from the sensors can be arranged in various sequences. The concatenation can be arranged based on the different randomness. Different sequences generated different concatenated images. Without losing the generality, the concatenation of RGB images was conducted to clearly show “spurious edges” by MTF transformation which has shown the better result in the Wafer dataset. 

**Figure 6:**
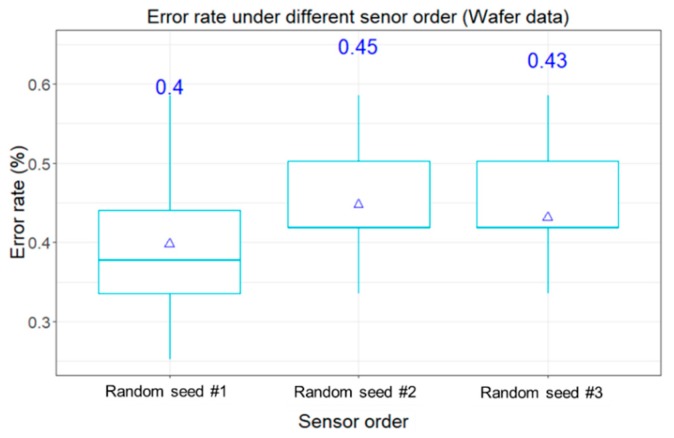
The boxplot of error rates of classification by using the MTF RGB images as inputs under different random sequences (Wafer dataset).

By following the same framework in Experiment #1, Figure 6 shows the box plot of 20 classification results under three different sequences that are based on different random number seed in the experiment. No matter which sequence was applied, the means of classification errors are around 0.4~0.45. The Wilcoxon Signed Rank Test was applied to check the pairwise comparison among these three random sequences. The statistical test also confirmed no significantly difference on the classification performance under the pairwise comparisons. It means the sequence of concatenating the images will not significantly influence the classification. This test also demonstrated that the ConvNet is able to learn image features regardless of the sequence of concatenation (or the patterns of edges).

#### 4.3.3. Experiment #3: Comparison of Different Architectures of ConvNet

**Figure 7:**
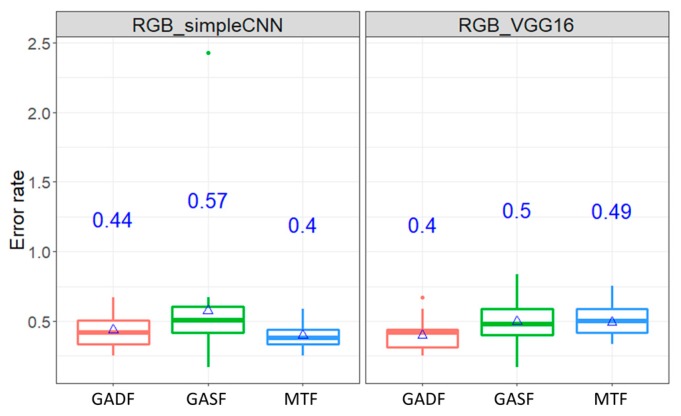
The boxplot of error rates of classifying RGB Wafer dataset images using simple ConvNet and VGG16.

**Figure 8:**
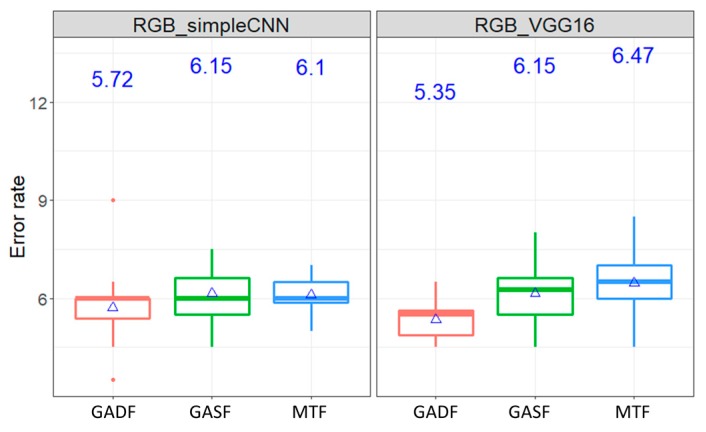
The boxplot of error rates of classifying RGB ECG dataset images using simple ConvNet and VGG16.

In the third experiment, two architectures of ConvNet: simple ConvNet and VGG16, were represented as the simple and complicated network structures, respectively. It is worth noting that VGG16 has the more complicate (deeper) network than simple ConvNet. Figure 7 shows that in the Wafer dataset, the average error rates under the simple ConvNet and VGG16 fall between 0.4% and 0.57%. The average error rates range from 5.35% to 6.47% in the ECG dataset, as shown in Figure 8. It can be seen, for each network structure, there is no significant different under different transformation methods. Further statistical analysis through the Kruskal–Wallis’s analysis of variance (Kruskal–Wallis ANOVA) proves that the error rates of these two ConvNet architectures are insignificantly different (*p*-value = 0.87 in the Wafer dataset and *p*-value > 0.999 in the ECG dataset). It simply means the complicated network structures does not necessarily guarantee better classification results. 

Table 3 shows the execution times of the simple ConvNet and VGG16 in processing the Wafer dataset and ECG datasets. It is obvious to show that VGG16 took more than ten times longer than the simple ConvNet in processing time, but the prediction accuracy improvement was insignificant. In short, the results of experiments show the interesting insights: encoding MTS data into colored concatenating image as inputs of the simple ConvNet can significantly improve the classification, however, the complicated network might not further improve it.

### 4.4. Comparison of Different Classification Tools

In literature, many methods were proposed to classify binary classes in Water and ECG MTS data. Table 4 enumerates the error rates conducted by different methods [4,37,38,39]. Please note that the average error rates are all limited to one-dimensional data transformation except our proposed methods starting with “concat”. As shown in this table, the proposed framework which uses three encoding methods with RGB by ConvNet produces better prediction accuracy in classifying Wafer and ECG datasets, indicated as bold face. In fact, the proposed concat-MTF-RGB can generate the best result (error rate = 0.4) in Wafer dataset while concat-GADF-RGB can obtain the best result (error rate = 5.35) in ECG dataset when comparing with previous works in literature. Therefore, once again, we can conclude that concatenating the encoded RGB images from multivariate time series data as the inputs of ConvNet following the proposed framework can significantly improve the classification accuracy, especially for the binary classification problems.

## 5. Conclusions

MTS classification tries to classify multiple univariate time-series data and predicts a class based on the learned patterns. This study proposed a framework of concatenating 2D images transformed from time series data as RGB input channels for ConvNet training. In this work, by following the convention, three image encoding methods: GASF, GADF, and MTF were used to encode MTS data into two-dimensional images after PAA dimension reduction. Then the MTS 2D images were concatenated as a big image separated by RGB channels to feed into ConvNet for binary classification. In order to investigate the impacts of: (1) the transformation methods, (2) the sequence of concatenation, and (3) the complexity of network structure on classification performance, a series of experiments were conducted. Three transformation methods, three different random sequences of concatenation (only for Wafer dataset), and two kinds of ConvNet architectures (simple ConvNet vs. VGG16), were used to assess the effects of these adjustments on the prediction accuracy.

Based on experimental results, the proposed framework applying the concatenated RGB images and with simple architecture of ConvNet can significantly improve the classification results. It is interesting that the selection of encoding methods does not affect the prediction outcome significantly. Also, the sequence of image concatenation is not significant for classification accuracy. These findings actually release the troublesome of choosing the image transformation method and the order of image concatenation. 

Besides, the experiment of conducting the two ConvNet (simple and complicated VGG16) show they produced insignificantly different results based on colored concatenating images as inputs. This “simple is enough” finding can enlighten MTS classification practitioners that always starting with the simple network rather than complicated one when applying deep learning methods on MTS classification problem. Again, the proposed framework with encoding images and simple ConvNet architecture was compared with other methods published in the past literature. The proposed framework produced promisingly the lowest error rates in both Wafer and ECG datasets where multivariate variables are inputs to classify binary class (normal vs. abnormal).

There are several future directions to further study the model. First, in this work, only one ConvNet was used for training data. Another framework which utilizes parallel ConvNets for each time series data and joins them in the last layer for prediction can be constructed. It would be worth evaluating if the parallel network will improve the accuracy. Second, developing a transformation method that can preserve both the dynamic and static information in the temporal range at the same time, or filter out irrelevant noise in the time series may be helpful to increase the feature distinctiveness in the images. Third, it might be interesting to check if more monochrome than RGB can improve the classification further. Last but not least, as the current framework was applied in binary classification datasets only, multiclass classification can be explored to assess the proposed framework performance.

## Figures and Tables

**Table 1:** Description of datasets.

Dataset	Instances	Variables	Classes	Min Length	Max Length
Wafer	1194	6	2	104	198
ECG	200	2	2	39	152

**Table 2:** The *p*-values of the Dunn Test in the RGB Wafer and ECG datasets images.

Comparison	*p*-Value	
	Wafer	ECG
GADF and GASF	0.487	0.116
GADF and MTF	0.061	0.201
GASF and MTF	0.018	0.947

**Table 3:** Average execution times (in seconds) of the simple ConvNet and VGG16 in processing the Wafer and ECG dataset images, separated by three data transformation methods.

Dataset	ConvNet Architecture	GADF	GASF	MTF
Wafer	Simple ConvNet	414.78	382.75	295.15
	VGG16	4619.19	5658.32	4805.03
ECG	Simple ConvNet	135.90	136.47	135.96
	VGG16	2356.57	2405.64	2358.46

**Table 4:** Comparison of average error rates (%) of different methods in Wafer and ECG datasets.

Approach	Wafer	ECG
DTW [4]	2.01	18.5
DDTW [4]	9.21	14
DDDTW [4]	1.92	14.5
STKG-SVM-K3 [37]	1.23	14.7
STKG-NB-K5 [37]	3.69	13.01
STKG-IF-PSVM-DT+M [37]	0.84	21.77
STKG-IF-NB-SVM+M [37]	2.23	9.71
normDTW [38]	3.85	16
combDTW [38]	2.01	16
LSTM-FCN [39]	1	15
MLSTM-FCN [39]	1	14
ALSTM-FCN [39]	1	14
MALSTM-FCN [39]	1	14
**concat-MTF-RGB** (ours)	**0.4**	6.1
**concat-GASF-RGB** (ours)	0.57	6.15
**concat-GADF-RGB** (ours)	0.44	**5.35**
